# Soybean resilience to drought is supported by partial recovery of photosynthetic traits

**DOI:** 10.3389/fpls.2022.971893

**Published:** 2022-10-20

**Authors:** Heba H. Elsalahy, Moritz Reckling

**Affiliations:** ^1^ Leibniz Centre for Agricultural Landscape Research (ZALF), Müncheberg, Germany; ^2^ Albrecht Daniel Thaer-Institute of Agricultural and Horticultural Sciences - Crop Science, Humboldt-University of Berlin, Berlin, Germany; ^3^ Botany and Microbiology Department, Faculty of Science, Assiut University, Assiut, Egypt; ^4^ Department of Crop Production Ecology, Swedish University of Agricultural Sciences (SLU), Uppsala, Sweden

**Keywords:** drought, leaf thermal recovery, photosynthetic recovery, resilience, soil moisture, soybean

## Abstract

Climate change affects precipitation dynamics and the variability of drought frequency, intensity, timing, and duration. This represents a high risk in spring-sown grain legumes such as soybean. Yet, under European conditions, no evidence supports the potential recovery and resilience of drought-tolerant soybean cultivars after episodic drought, at different growth stages. A field experiment was conducted using a representative drought-tolerant cultivar of soybean (cv. Acardia), in 2020 and 2021, on sandy soils in Germany, applying four water regimes (irrigated, rainfed, early-drought, and late-drought stress). Drought stress was simulated by covering the plots during the event of rain with 6 × 6 m rainout shelters, at the vegetative (V-stage) and flowering (Fl-stage) stages. Drought response was quantified on plant height, chlorophyll fluorescence ratio (ChlF ratio), chlorophyll content (Chlc), and leaf surface temperature (LST), at different intervals after simulating drought until pod filling. Grain yield and yield components were quantified at the end of the growing season. Compared to rainfed conditions, a drought at V-stage and Fl-stage reduced significantly plant height, ChlF ratio, and Chlc by 20%, 11%, and 7%, respectively, but increased LST by 21% during the recovery phase. There was no recovery from drought except for Chlc after V-stage in 2021, that significantly recovered by 40% at the end of the growing season, signifying a partial recovery of the photochemical apparatus. Especially, there was no recovery observed in LST, implying the inability of soybean to restore LST within the physiological functional range (**Graphical abstract**). Under rainfed conditions, the grain yield reached 2.9 t ha^-1^ in 2020 and 5.2 t ha^-1^ in 2021. However, the episodic drought reduced the yield at V-stage and Fl-stage, by 63% and 25% in 2020, and 21% and 36% in 2021, respectively. To conclude, the timing of drought was less relevant for soybean resilience; however, pre- and post-drought soil moisture, drought intensity, and drought duration were likely more important. A drought-tolerant soybean cultivar may partially be drought-resilient due to the recovery of photosynthetic traits, but not the leaf thermal traits. Overall, these findings will accelerate future efforts by plant breeders, aimed at improving soybean drought resilience.

## Graphical Abstract

Experimental design for soybean (Glycine max L.; cv. Acardia) grown in a field experiment at ZALF under four water regimes (irrigated, rainfed (control), early-drought, and late-drought stress), and main results on resilience to drought for Chlorophyll fluorescence ratio (ChlF ratio), Chlorophyll content (Chlc), leaf surface temperature (LST), and grain yield during the vegetative stage (V-stage) and flowering stage (Fl-stage).

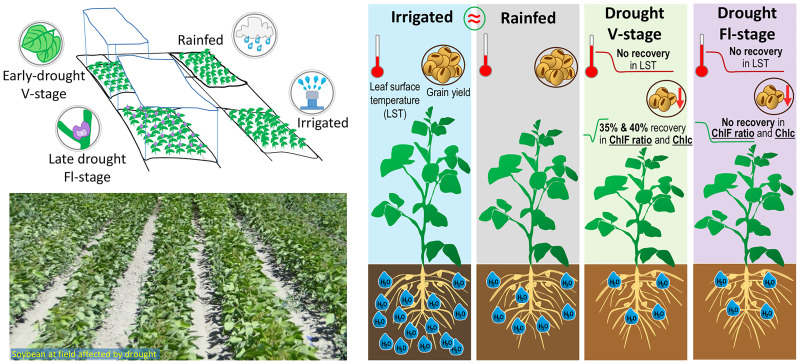

## Introduction

Climate models predict an increase in the annual air temperature in Central Europe by 3.6-6°C in summer, while precipitation is projected to decrease by 7-20% at the end of the 21^st^ century (Coppola et al., 2021; Politi et al., 2022). However, in the present day, high variability of drought frequencies, intensities, timing, and duration has been observed across various European climate regions with extreme drought events in Central Europe (Beillouin et al., 2020; IPCC, 2021). This situation is especially insecure for spring-sown grain legumes such as soybean, which have a tendency for less stable yields than winter crops (Reckling et al., 2018). To face this problem, plant scientists and breeders worked hard to hunt drought-tolerant genotypes and document yield-related traits that back cultivar growth and productivity (Chen et al., 2016; Guzzo et al., 2021). They tested the cultivars mostly through a scenario called cumulative drought stress, which simulates a condition of low frequent precipitation during the season (Elsalahy et al., 2020; Gao et al., 2020; Saleem et al., 2022). Based on the findings, the researchers claimed that drought-tolerant cultivars are a promising candidate for building resilient crop production systems (Arya et al., 2021; Guzzo et al., 2021; Monteoliva et al., 2021; Arab et al., 2022). Whereas, within an agriculture context, the change in rainfall pattern can cause episodic drought, which is a prolonged period of no precipitation that is projected to occur more frequently in the future (Hari et al., 2020). Yet, there is no empirical evidence to prove that the drought-tolerant cultivars can recover from an episodic drought or prolonged shortages in precipitation. In addition, most studies that evaluated the response of tolerant cultivars to drought were conducted in greenhouses and chambers, which may be unspecific due to excluding most environmental variations (Dong et al., 2019; Lotfi et al., 2019; Sakoda et al., 2021). Hence, there is a need to study the response of the tolerant crops/cultivars to a periodic or episodic drought event, under field conditions, which may show specific responses, judged by the capacity of the considered cultivar to recover. So far, it is not well understood how tolerant crops/cultivars would recover from current or future drought events and to what extent would be their contribution to building a resilient agriculture system.

Soybean (*Glycine max* L.) is worldwide the most cropped legume, as it represents a vital source of vegetable protein for both humans and livestock (Wang et al., 2022). Soybean is sensitive to low soil moisture, however, it is adapted to a large range of temperatures, and its cultivation is extended toward Northern Europe (Boulch et al., 2021; Karges et al., 2022). Despite that, little is known about the soybean response and resilience capacity to various drought events at different growth stages, under European conditions. Primarily, the response of soybean to drought is conditioned by the duration and intensity of the stress, as well as the growth stage when the stress occurs (Wei et al., 2018; Dong et al., 2019; Yan et al., 2020; Cui et al., 2021; Saleem et al., 2022). During the vegetative stage, soybean water requirements are low but reach a maximum during the flowering to pod filling stage, and then decline as the plant matures (Norberg et al., 2010; Pejić et al., 2011; Gajić et al., 2018; Lamichhane et al., 2020). Mild drought stress, during the vegetative stages, can reduce soybean growth (plant height), and grain yield, but if the stress ends at this stage, soybean could compensate for water stress (Gajić et al., 2018; Wei et al., 2018; Sakoda et al., 2021; Wang et al., 2022). Conversely, any grade of drought, during the flowering to pod setting stage, can significantly cause irreversible negative effects on soybean growth and yield (Wijewardana et al., 2018; Ismanov et al., 2019; Boulch et al., 2021; Pinnamaneni et al., 2021). Understanding soybean response and recovery dynamics after drought would contribute to pragmatic implications for remedies (e.g., breeding to improve traits), and management (e.g., irrigation).

Mostly, the damage caused by drought at any stage of soybean growth is primarily attributed to the inhibition and disruption of photosynthesis, which is the fundamental process for maintaining plant growth and recovery after releasing drought (Wang et al., 2018; Iqbal et al., 2019; Sakoda et al., 2021). Photosynthetic-related traits, particularly chlorophyll content, have been studied for decades to screen genotypes for drought tolerance (Guzzo et al., 2021; Monteoliva et al., 2021; Sakoda et al., 2021). The findings of these studies showed that drought-tolerant cultivars have higher chlorophyll contents or are at least able to maintain chlorophyll content under drought, by that having the potential for higher photosynthetic rate and yield (Chen et al., 2016; Guzzo et al., 2021; Monteoliva et al., 2021). To prepare for the drastic and rapid change in the global climate, measuring chlorophyll content was adopted as a quick measurement for selecting tolerant cultivars (Guzzo et al., 2021; Monteoliva et al., 2021). Further, thanks to the high-throughput technologies, measuring chlorophyll content can be repeated in the same sampling area to assess temporal dynamics during plant growth. This technical possibility of measuring chlorophyll content is essential for monitoring plant recovery and resilience to drought dynamics.

Moreover, the chlorophyll fluorescence technique is a valid non-destructive physiological indicator that has been used to monitor the photosynthetic capacity under drought stress (Wang et al., 2018; Dubberstein et al., 2020; Zhuang et al., 2020; Arab et al., 2022). The chlorophyll fluorescence ratio, Fv/Fm, which is the maximum photochemical efficiency of Photosystem II (PSII), is the parameter that has been widely used as an index of drought-induced injury in leaves (Wang et al., 2018; Trueba et al., 2019; Zhuang et al., 2020). Mainly, the chlorophyll fluorescence ratio can provide detailed information about the status and function of PSII, reflecting the plant’s ability to collect and transfer light energy, hence, the plant’s ability to tolerate or resist abiotic stress (Wang et al., 2018; Zhuang et al., 2020). Drought reduces the chlorophyll fluorescence ratio ascribed by the underutilization of light energy absorbed by PSII, inferring the down-regulation of photosynthesis or photo-inhibition (Buezo et al., 2019; Dubberstein et al., 2020; Zhuang et al., 2020). The chlorophyll fluorescence ratio is largely and positively related to chlorophyll content under drought stress (Wang et al., 2018; Zhuang et al., 2020). Studies proved that the light absorption and fluorescence emission depend on the concentration of chlorophyll molecules in the chloroplast; thus, the decrease in chlorophyll content causes weakness in the photochemical process, leading to a decrease in the chlorophyll fluorescence ratio (Dubberstein et al., 2020; Zhuang et al., 2020). In this context, exploring the dynamics of fluorescence parameters and chlorophyll content under different drought scenarios may explain plant recovery after releasing drought; hence determining crop resilience capacity in face of unpredicted climate change.

Besides, drought-tolerant soybeans have adaptive traits to overcome the adverse effects of drought, such as decreasing stomatal opening, which is a mechanism associated with reducing photosynthesis ascribed to the decrease in carbon dioxide sink (Wang et al., 2018; Trueba et al., 2019; Sakoda et al., 2021; Arab et al., 2022). Stomatal closure leads subsequently to a decrease in transpiration, followed by an increase in leaf temperature above the optimal level. This increase in leaf temperature affects the activity of photosynthetic enzymes such as Rubisco, leading to down-regulation and/or photodamage in PSII, hence lower photosynthesis (Iqbal et al., 2019; Dubberstein et al., 2020; Moore et al., 2021). In this context, the magnitude at which the leaf can cool down to keep the leaf temperature within a physiological functional range may identify the thermal capacity of the plant to adapt to changes in climate. In soybean, maintenance of leaf temperature has been reported to be highly related to the internal water status of the plant. Therefore, leaf cooling has been suggested as a useful trait in identifying plant acclimation to drought (Deva et al., 2020; Moore et al., 2021; Saleem et al., 2022). In this context, quantifying the recovery of leaf temperature after releasing drought is needed to provide evidence of whether leaf or canopy cooling can contribute to the resilience of soybean to drought.

So far, we don’t know whether drought-tolerant cultivars of soybean can be resilient to episodic drought under field conditions. Second, we asked which of the studied traits, photosynthetic or leaf thermal traits, would be highly linked to soybean resilience. Third, to consider future remedies and management strategies, it was important to specify at which growth stage soybean fails in drought resilience. Finally, to ensure the sustained yield of soybean, it was much needed to determine the extent to which soybean resilience can be reflected in the yield. Therefore, we conducted two field experiments in the seasons 2020 and 2021 and applied four water regimes on soybean comprising irrigated, rainfed, early-drought (V-Stage), and late-drought (Fl-stage) stress. Soybean responses and resilience to episodic drought were quantified on plant height, chlorophyll fluorescence ratio (ChlF ratio), chlorophyll content (Chlc), and leaf surface temperature (LST), at different intervals after simulating drought until pod filling. Grain yield and its components were estimated at the end of the season. Using these responses, we tested key hypotheses: (1) Soybean recovery, in terms of the measured metrics, is greater when drought is short and/or less intense. (2) The potential recovery of soybean after releasing the episodic drought is greater when drought occurs during V-stage than drought during Fl-stage. (3) Both the photosynthetic and leaf thermal traits are equally linked to soybean resilience; and (4) the recovery of the considered traits would be strongly reflected in the yield.

## Materials and methods

### Cultivar, site, and climate characteristics

To measure the potential recovery and resilience of soybean to episodic drought, one representative, new, highly promising drought-tolerant cultivar (cv. Acardia) was used. The source of this cultivar is SAATEN-UNION GmbH, Germany. Our study presents, for the first time, the morphological, photosynthetically, and leaf thermal characteristics of the considered cultivar, under our field conditions in Northern Germany. Specifically, Acardia is a medium early maturing cultivar (within the maturity group 000), that was described to have a high grain and protein yield, high TGW, light navel, and good resistance to sclerotinia (Hofmann et al., 2019). Further, acardia is suitable for human consumption and animal feed. Importantly, Acardia is a cultivar with above-average performance, especially under northern and eastern European conditions (Reckling, 2022; Sidorova, 2022). Precisely, in official cultivar trials in Bavaria, Acardia produced high grain yields because of the good stability in combination with a high set of lower pods, which ensured low harvest losses (Hofmann et al., 2019). Therefore, Acardia was described as a climate-resilient cultivar. To adapt to the current and future climate change, it is important to investigate the performance of the Acardia cultivar at another site to assess its productivity across different pedoclimatic regions of Europe.

Two field trials were conducted in the same field, in the seasons 2020 and 2021. Both trials were positioned at the experimental station of the Leibniz Center for Agricultural Landscape Research (ZALF) in Müncheberg, 40 km east of Berlin, Germany (52° 31′ 2.121′′ N, 14° 07′ 30.453′′ E, 62 m a.s.l.). The soil in the field trial is sandy (Eutric Cambisol) with an average content of sand, silt, and clay of 83%, 9%, and 8%, respectively, at a depth of 0-30 cm (Huynh et al., 2019). The water table is approximately 12 meters below the surface. It should be noted, however, that there may be deviations within the test areas, as the soil conditions are very heterogeneous. In the previous years of conducting the current experiment, the field was used for an experiment on maize production (Huynh et al., 2019).

According to the meteorological data on the experimental site (ZALF, Müncheberg, Germany), the climate from 1981 to 2010 was characterized by high temperatures in late spring and summer, frequent dry periods in early summer, and cold winters with mostly little snow. Specifically, the mean annual air temperature was 9.5° C; mainly, the mean winter and summer temperatures were 0.8° C and 18.2° C, respectively. The mean annual precipitation was 565 mm with the most precipitation in the summer, on an average of 217 mm, but the mean winter precipitation was 98 mm. Particularly, the highest precipitation and temperature values were recorded in July, and the highest global radiation and the lowest relative humidity were recorded in June. The mean annual global radiation was 13.2 MJ m^−2^; the mean winter and summer radiations were 6.2 MJ m^−2^ and 20.7 MJ m^−2^, respectively.

### Experimental design

The experimental design was implemented in one plot of 9 × 35 m. Within this plot, four sub-plots were arranged that occupied the same area of 6 × 6 m in both years. Each sub-plot was divided internally into three blocks to allow replication of the sampling. The area of the rainout shelter was 6 × 6 m, newly used in the experimental site, and installed at a height of 1.5 m above the soil surface (see pictures **Supplementary Figure S1**). We had only pseudo-replicates in our experiment. True replicates were not possible as we only had two rainout shelters available due to the high costs and area demand. While true replicates would be theoretically possible with smaller mobile shelters (see e.g., Kundel et al., 2018 describing 2.5 m x 2.5 m shelters) but these usually have much more side effects and cannot simulate drought as precisely as our large 6 m x 6 m shelters that included barriers to stop water running into the plots. Hence, there is a trade-off between true replication and precision of the simulated stress. We still aimed to account for the variation within one plot by having pseudo-replicates. Also, it was essential to evaluate the accuracy of the used shelter in creating differences in soil moisture before considering using more shelters to screen many cultivars at the same time. The shelters were closed manually during the event of rain during the selected periods and then reopened after the rains stopped to avoid any effects of the roof on temperature, evaporation, etc.

Four water treatments were established in the two experiments. 1) Irrigation treatment: called *irrigated*, in which the plots were irrigated with 20 mm water at different intervals using a unique irrigation system simulating rainfall (**Supplementary Figure S1**). The amount of irrigated water was determined by the WEB-BEREST model that calculates the irrigation water based on the crop demand using the coefficient of actual to potential evapotranspiration (Mirschel et al., 2020). The adopted irrigation strategy resulted in an amount of irrigated water of 280 mm (14 times) and 140 mm (7 times) in 2020 and 2021, respectively (**Supplementary Table S1**). 2) Rainfall treatment: called *Rainfed*, where the plots received only precipitation. 3) Early drought stress: called *early drought*; simulated spring-summer drought spell, in which the plots were sheltered with a roof at the rapid vegetative growth (V-stage; BBCH 15–17, between V3 and V5, where V3 and V5 are defined when soybean plants display three and five fully expanded trifoliate leaves, respectively; Sobko et al., 2019). The drought event during the V-stage was implemented for 24 days (3^rd^ June–27^th^ June) and 14 days (11^th^ June–25^th^ June) in 2020 and 2021, respectively. 4) Late drought stress: called *late drought*; simulate summer drought spell, in which the plots were sheltered at the flowering stage (Fl-stage; BBCH 61–69; the beginning of flowering–end of flowering: the first pod is visible, approximately 5 mm in length). This treatment was implemented for 26 days (28^th^ June–24^th^ July) and 29 days (26^th^ June–25^th^ July) in 2020 and 2021, respectively (**Supplementary Figure S2**).

### Data collection: soil, air, and plant parameters

The field was prepared with a cultivator ploughed in spring and the seedbed was prepared before sowing. Soybean was sown at a depth of 3-4 cm on the 4^th^ and 6^th^ of May in 2020 and 2021, respectively, when soil temperature was constantly above 10°C. Each plot contained 24 rows with 37.5 cm spacing between the rows. The sowing density was 70 seeds m^-^² as this density (Sobko et al., 2019). Seeds were inoculated with HISTICK^®^ (BASF, Germany), a peat-based product that showed affective nodulation under similar bio-physical conditions (Reckling et al., 2020). The field was not fertilized during the years of the experiment, and soybean was treated with a pre-emergent herbicide to control weeds (no other pesticides were applied).

Soil moisture was measured at 10 cm depth by using sensors (EC5 sensors from METER Group, Inc. USA) that were installed at three points within each sub-plot (per treatment). These points were randomized to avoid edge effects, such as runoff of rainwater from neighboring plots. The sensors recorded the soil moisture (volumetric water content; VWC; m^3^ m^-3^) every 15 minutes and were read out every two weeks. Further, soil moisture and soil temperature were measured, at five points within each sub-plot, at two soil depths (30 and 60 cm), by using the soil hydrological measuring stations from the company Umwelt-Geräte-Technik GmbH (UGT DL200). These data loggers provided also values every 15 minutes and were read out every two weeks. In addition, the air temperature and air humidity were measured at three heights (10, 50, and 100 cm) using HOBO MX2301 data loggers (measuring every 15 minutes during the entire growing period).

Four plant parameters were chosen that comprised plant height, chlorophyll fluorescence ratio (ChlF ratio), total chlorophyll content (Chlc), and leaf surface temperature, and measured, in three replicates, 8 times in 2020 and 7 times in 2021, during the growing season. These parameters were selected intentionally to meet the need for rapid, non-destructive, repeated measurements, under field conditions, to create time series data that allows an understanding of the dynamic of resilience over the entire growing season. Chlorophyll fluorescence ratio and total chlorophyll content were measured in the leaves using a portable chlorophyll content meter (CCM 300 sensor, optic-sciences, USA) that works with a proven chlorophyll fluorescence ratio of F735 nm/F700 nm. Chlorophyll was measured at the first fully developed leaf at the top of the plant. Leaf surface temperature was measured with the VOLTCRAFT (IR-2200-50D) thermometer.

At the end of the soybean growing season, to determine yield structure, 0.5 linear meters were cut by hand into six replicates per plot, i.e., 0.19 m² per cut (0.5 m × 0.375 m). The harvest was done on the 22^nd^ and 28^th^ of September, in 2020 and 2021, respectively. Six parameters were measured at soybean harvest that included plant height (cm), grain yield (t ha^-1^ at 100% dry matter), thousand-grain weight (g), number of secondary branches per plant, pod number per plant, and seed number per plant.

### Calculation of indices

To quantify resilience, we used equation 6 in Elsalahy et al. (2020) according to Orwin and Wardle (2004).


(1) $$r\text{ = }\frac{{2|D}_{0}|}{{\text{(|D}}_{0}{\text{|+ (|D}}_{\text{x}}\text{|) }}-1$$

Where *D_0_* is the difference between the selected response variable of the rainfed treatment and the stressed plants at the end of the drought event at (t_0_). And D_x_ is the difference between the rainfed treatment and the stressed plants at the time point t_x_ chosen to measure resilience (recovery). This resilience index *r* is bounded by -1 and +1, with maximal resilience at +1. This index is standardized by the amount of change initially caused by the drought (D_0_), as this determines the state from which it has to recover.

### Statistical analysis

The four plant parameters comprising plant height, ChlF ratio, Chlc, and LST were non-normally distributed according to the Shapiro-Wilk test. The variances between the groups were homogeneous according to Levene’s test in the *car* R-package (John and Weisberg, 2011). As a remedy for the pseudo-replicates in our experiment, while performing the statistical analysis, we used Mixed-effects models that allowed using the concept of random effects to emulate the randomness inherent in the data (Lazic, 2010; Lazic et al., 2020). In addition, we averaged the dependent data points in each plot and compared the average of the four treatments at each point in time. The linear mixed model (lme) was used in the *nlme* package (Pinheiro et al., 2021) and the autocorrelation function (ACF) was used to estimate the correlation between the measured values in the time series. To set the correlation structure in the model, the corAR1 correlation function was added with the *Time* variable nested in the year variable as a covariate. To adjust the variance structure, the varIdent function for the combination of the variables *Yr* and *Trt* was added to the model. The selected model was compared with different models in the *MuMIn* R-package (Barton, 2020), and according to the AIC (Burnham et al., 2011), it showed the lowest AIC value (**Supplementary Table S2**). To create consistent comparisons among the measured parameters, the same model was used, including three independent variables representing *Trt* (treatment), *Time* (date when the different plant parameters were measured), *Yr* (year), and the interaction between them. As a further step, to provide a more straightforward interpretation of the differences among the water treatments in each year, the data for each year were analyzed separately as sub-models with the two factors *Trt, Time, and Trt x Time*. In each sub-model, residuals ‘normality and variance homogeneity were checked.

This resilience index, which compares the absolute difference that exists between post-drought and control treatment (Rainfed) relative to the initial absolute effect of the drought, was first evaluated by considering the variables *Yr*, *Trt*, *and Yr* x *Trt*. After ANOVA, Tukey’s HSD test at α = 0.05 was used to determine the significance of differences among the mean values of the *Trt* (early drought and late-drought) at each given point of time by using the *Agricola* package (De Mendiburu, 2019).

Linear regression models were used to investigate the yield structure parameters, as the data of these measurements were normally distributed and the variance was homogeneous. The first model included two independent variables representing *Trt*, *Yr*, and *Trt* × *Yr*. Afterward, sub-models were used to evaluate the difference between the four water treatments every year. The block effect was not significant and when added to the model, this showed no improvement in the primary model according to AIC (Burnham et al., 2011); therefore, it was removed in all sub-models to create consistent comparisons among the models. All statistics were performed using R (version 4.1.1) with R studio (version 2021.09.0) (R Core Team, 2021).

## Results

### Weather differences in the crop seasons

**Figure 1:**
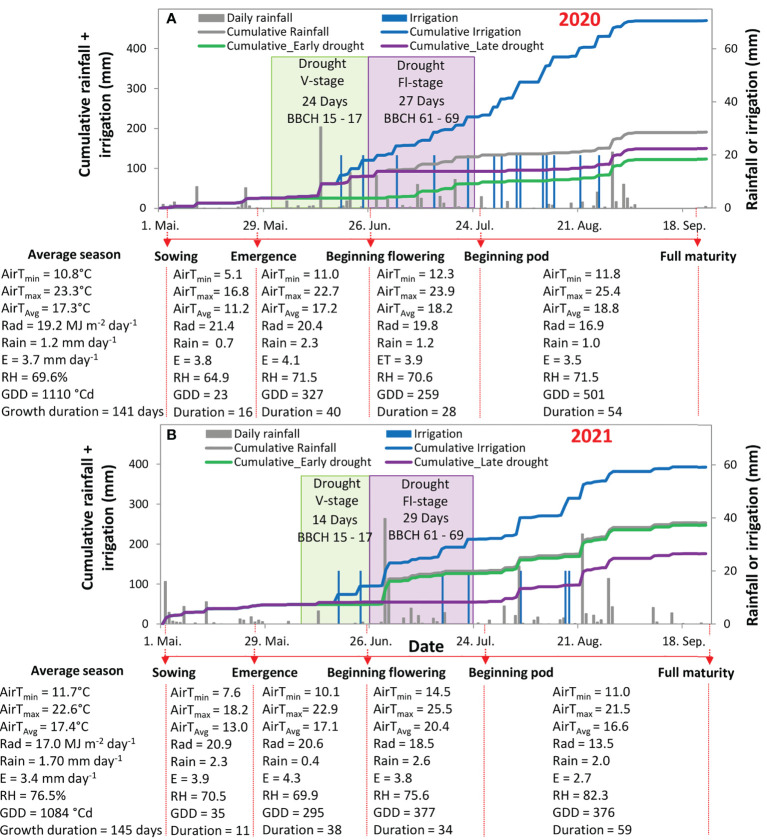
Cumulative water input (rainfall + irrigation; left axis; **A, B**) and daily rainfall or irrigation (right axis; both panels) from the beginning of May till the end of September in the season 2020 **(A)** and 2021 **(B)**. The green shaded area indicates the duration of using a shelter above the plants at the vegetative stage (V-stage) and the violet shaded area indicates the duration of using a shelter above the plants at the flowering stage (Fl-stage). The Meteorological elements and bioclimatic index for the soybean development stages, in both years, are in the tables below each figure specifying, in each phenological period, the minimum temperature (T_min_), the maximum temperature(T_max_), the average temperature (T_Avg_), the average solar radiation (Rad), the average rainfall (Rain), the daily evaporation (E), the percentage of relative humidity (RH), and the growing degree days (GGD).

**Figure 2:**
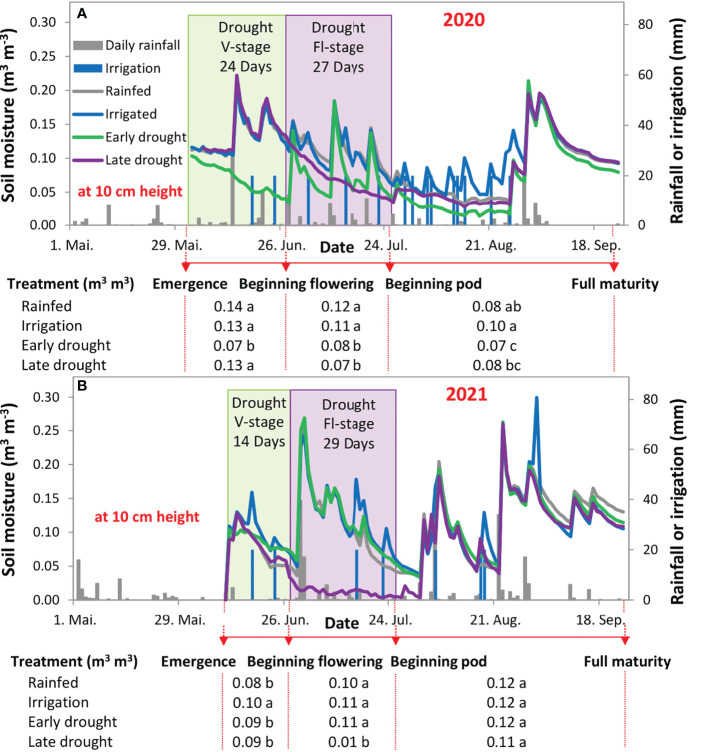
Soil moisture at 10 cm height (left axis; **A, B**) and daily rainfall or irrigation (right axis; both panels) from the beginning of May till the end of September in 2020 **(A)** and 2021 **(B)**. The green shaded area indicates the duration of using a shelter above the plants at the vegetative stage (V-stage) and the violet shaded area indicates the duration of using a shelter above the plants at the flowering stage (Fl-stage). The Meteorological elements and bioclimatic index for the soybean development stages, in both years, are in the tables below each figure.

Overall, precipitation, soil moisture, and solar radiation significantly differed between the two cropping seasons (May-September) in 2020 and 2021, but not the air temperature (**Figures 1**, **2**). The amount of precipitation from May to September reached 177 mm in 2020 and 254 mm in 2021, which resulted in a dry season in 2020 with 25% less rainfall than in 2021. Specifically, during the early stage of the growing season from sowing to seedling growth (1st May–end of May), the amount of rainfall reached 13.5 mm in 2020 vs. 38.8 mm in 2021, representing 65.2% less rainfall during the seedling development in 2020 than in 2021 (**Figures 1A, B**). During the vegetative stage, the rainfall amount reached 65.0 mm in 2020 *vs.* 16.2 mm in 2021 (see dates in **Figures 1A, B**). This was mainly because of two heavy rainfall events in 2020, with 30.5 mm and 13.2 mm rainfall (**Figures 2A, B**). In contrast, during the flowering stage, the plants received less rainfall in 2020, representing 41.1 mm, and higher amounts in 2021, representing 77.7 mm. Noticeably, the amount of rainfall in 2021 during the flowering stage was because of a heavy rain event that occurred immediately at the beginning of this stage for two successive days and reached 39.7 and 17.2 mm per day (see dates in **Figure 1**). The amount of precipitation that coincided with the beginning of the pod until full maturity (July to September) differed significantly (p < 0.001) between the two seasons and showed 57.1 mm in 2020 vs. 121.1 mm in 2021 (**Figures 1A, B**). Despite the temporal variation in rainfall patterns in both years, the rainfall distribution was more balanced in 2021 than in 2020.

The soil moisture at 10 cm depth was significantly (p < 0.001) different during the two cropping seasons and between the four water regimes (**Figures 2A, B**). In 2020, at the V-stage, the simulated drought treatment showed a significant reduction in soil moisture by 50% in comparison with the other treatments (**Figure 2A**). However, in 2021, at the same phenological stage, no difference was observed between the rainfed and drought treatment (**Figure 2B**). During the Fl-stage, in 2020, soil moisture differed significantly for both the early and late drought treatments and showed lower values by 30% and 40%, respectively, in comparison with the average of rainfed and irrigation treatments. In comparison with the same phenological stage in 2021, only the late drought treatment showed a reduction in soil moisture by 91% in comparison with the other treatments (**Figure 2B**). At the late stage of the crop cycle (beginning of maturity until harvest), soil moisture was, in 2020, significantly higher at the irrigated treatment by 20-30% in comparison with the rainfed and the two drought treatments (**Figure 2A**). However, in 2021, no differences in soil moisture were recorded between the different treatments (**Figure 2B**). The difference was lower in soil moisture, between the four water regimes, at soil depths of 30 and 60 cm (**Supplementary Figure S3**).

The difference in solar radiation between the two seasons was highly significant (p < 0.001) with an average of higher value in 2020 than in 2021 (**Figures 1A, B**). Namely, in 2020, the solar radiation from sowing till the end of the Fl-stage was 19.5 MJ m^-2^ day^-1^ and dropped afterward to 16 MJ m^-2^ day^-1^ (**Figure 1A**). The same trend was also observed in 2021, but with lower values that reached an average of 17.7 MJ m^-2^ day^-1^ till the end of flowering and dropped afterward to 13.5 MJ m^-2^ day^-1^ (**Figure 1B**). The average temperature between the two growing seasons was comparable; however, a significant (p < 0.001) difference was observed, in average temperature, during the maturity stage (end of July to end of September) with an 11% lower temperature in 2021 than in 2020 (**Figure 1B**). As a result, the growing degree days were, on the average of the four water regimes, 1125°Cd in 2020 vs. 1000°Cd in 2021 (**Supplementary Figure S4**).

### Soybean growth, photosynthetic traits, leaf temperature at different growth stages under different water regimes

**Figure 3:**
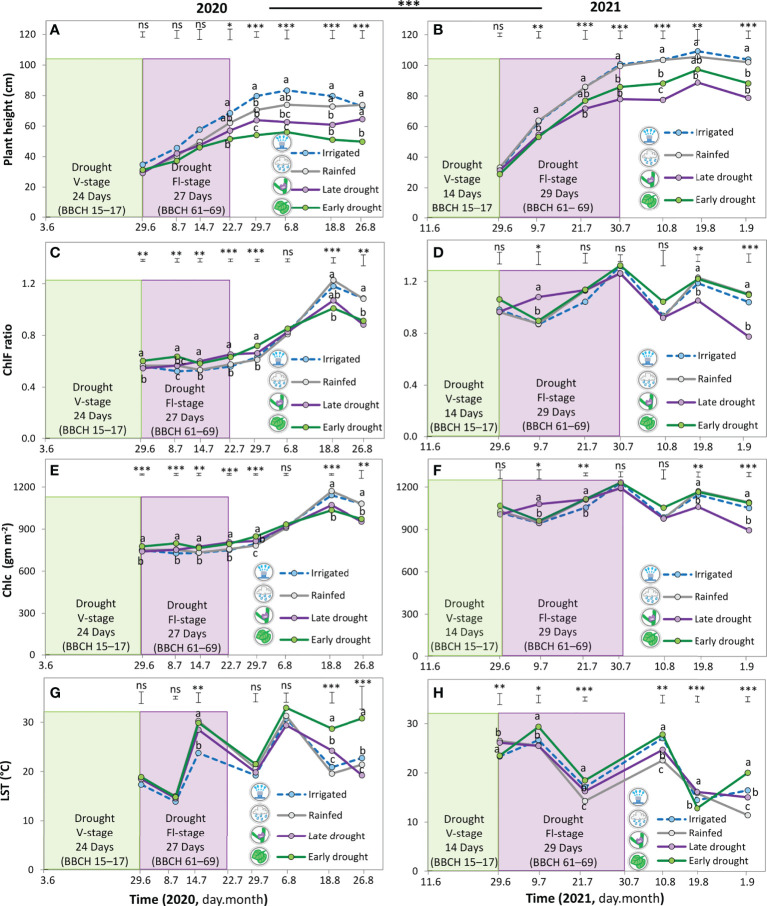
Plant height **(A, B)**, chlorophyll fluorescence ratio (CFR; **C, D**), chlorophyll content **(E, F)**, and leaf surface temperature **(G, H)** of soybean under four water treatments, 3 drought scenarios + the control (irrigated) treatments in 2020 **(A, C, E, G)** and 2021 **(B, D, F, H)**. The green shaded area indicates the duration of the early drought event at the vegetative stage (V-stage) and the violet shaded area indicates the duration of the late drought event at the flowering stage (Fl-stage). The vertical bars represent Tukey’s HSD test (p < 0.05) at a given point in time (n = 3). Asterisks above the vertical bars indicate significant differences among the mean of the different treatments when ANOVA result was significant at P < 0.05; ***P < 0.001, **P < 0.01,*P < 0.05, ns; not significant.

On average, of the four water regimes, a significant (p < 0.001) reduction was observed in the measured parameters in 2020 than in 2021. Namely, plant height, ChlF ratio, and Chlc were reduced by 28%, 31%, and 19%, respectively, but an increase in LST by 24% (**Figures 3A–H** **;** **Supplementary Table S3**). Strikingly, in both years, there was no significant difference between the irrigation and rainfed treatments in any of the measured parameters. In comparison with the rainfed treatment, plant height was reduced after a drought during V-stage by 20% in 2020 and 13% in 2021. In contrast, after drought during Fl-stage, the reduction in plant height was 10% in 2020 and 19% in 2021 (**Figures 3A, B**).

In 2020, in comparison with the rainfed treatment, the temporal change in Chlc and ChlF ratio showed a significant increase of 4-8% during and post-drought at V-stage and Fl-stage (**Figures 3C, E**). However, in the same year, during the pod filling and maturing stage, from mid-August to the beginning of September, a sudden significant drop occurred in Chlc and ChlF ratio by 10% and 16% (**Figures 3C, E**). In 2021, the same trend was almost observed after a drought during V-stage and Fl-stage, but the increase in Chlc and ChlF ratio was not significant and represented around 3-5% (**Figures 3D, F**). However, in the same year during the maturity stage, the amount of Chlc was maintained after a drought during V- stage but dropped significantly in the treatment of drought during Fl-stage (**Figures 3D, F**).

The dynamics of change in LST did not differ significantly, in comparison with the rainfed treatment, when the plants were adept at sustaining or increasing Chlc and ChlF ratio (**Figure 3E, H**). This trend was notable in 2020, where no change was observed in LST after drought during the V-stage and Fl-stage until the beginning of the maturity stage. Afterward, the LST increased significantly, coincidently with the drop in Chlc and ChlF ratio, by 45% and 6%, after drought during V-stage and Fl-stage, respectively (**Figures 3E, G**). In 2021, in comparison with rainfed treatments, the LST increased significantly during the entire season after a drought during V-stage and Fl-stage by 9% and 11% respectively (**Figure 3H**).

### Resilience index for soybean growth, photosynthetic traits, LST after drought at V-stage and Fl-stage

**Figure 4:**
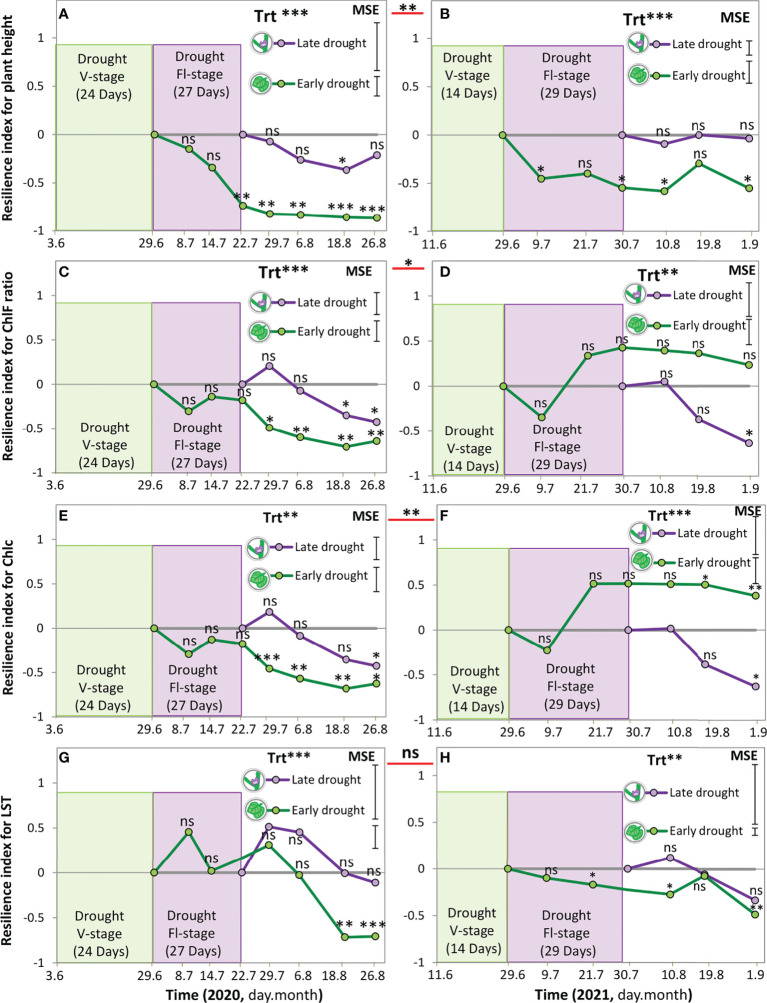
Resilience index for plant height **(A, B)**, chlorophyll fluorescence ratio **(C, D)**, Chlorophyll content **(E, F)**, and leaf surface temperature **(G, H)** of soybean under four water treatments, 3 drought scenarios + the control (rainfed) treatments in 2020 (A,C,E, and G) and 2021 **(B, D, F, H)**; equation 1. The green shaded area indicates the duration of the early drought event at the vegetative stage (V-stage) and the violet shaded area indicates the duration of the late drought event at the flowering stage (Fl-stage). The vertical bars represent Tukey’s HSD test (p < 0.05) of the mean standard error (n = 3). Asterisks beside the factor treatment (Trt) indicate significant differences between the resilience at early drought and late drought based on ANOVA result; *** *P* < 0.001, **P < 0.01, *P < 0.05, ns; not significant.

Overall, the resilience index revealed less resilience for soybean in 2020 than in 2021, by showing significant negative values for plant height, ChlF ratio, and Chlc, but not for LST (**Figures 4A–H**; **Supplementary Table S4**). Specifically, for plant height, after releasing the drought at any of the growth stages, the difference between the rainfed and drought treatments increased, leading to negative values in the resilience index, until the end of the season (**Figures 4A, B**). An exception for this observation was in 2021 when the resilience index after a drought during Fl-stage showed no change (almost a constant value of “0”); however, it was still no recovery in plant height in this case (**Figure 4B**).

For the photosynthetic traits; ChlF ratio and Chlc, the dynamics of recovery for these parameters were significantly different between the two years (p < 0.01) and between drought during V-stage and Fl-stage (p < 0.05) (**Figures 4C–F**). Only in 2021, the resilience index for drought during V-stage showed an increase in the ChlF ratio of 0.35 ± 0.23 and a significant increase in Chlc of 0.40 ± 0.27. This finding points out a 35% recovery in the ChlF ratio and a 40% recovery in Chlc, in comparison with the rainfed treatment (**Figures 4D, F**).

The resilience index for LST showed different dynamics between the two years. Specifically, in 2020, immediately after releasing the drought that occurred at the early and late growth stages, soybean showed a tendency for fast recovery. Positive values of the resilience index were observed that reached a maximum peak of 0.43. However, the capacity for continuous recovery decreased afterward and reached negative values for both early and late drought by -0.73 and -0. 05, respectively (**Figure 4G**). Conversely, in 2021, the resilience index for LST did not show any potential recovery after releasing drought. Instead, it was decreasing continuously until reaching a significant value for the early drought treatment that reached (**Figure 4H**).

### Yield components of soybean in response to drought under different water regimes

**Figure 5:**
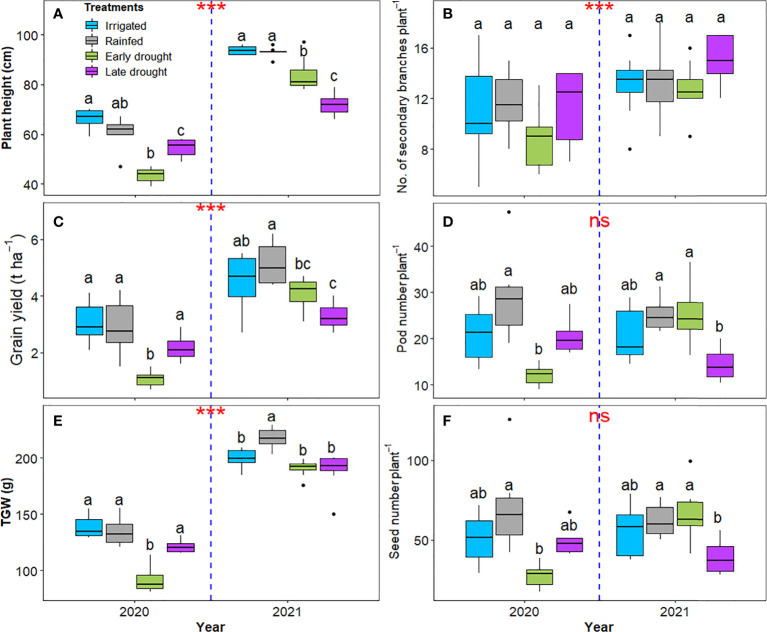
Boxplot of yield structure of soybean under four water treatments in 2020 and 2021. Plant height **(A)**, number of secondary branches per plant **(B)**, grain yield **(C)**, pod number per plant **(D)**, TGW **(E)**, and seed number per plant **(F)**. The different letters above the boxplots represent Tukey’s HSD test (p < 0.05) among the different treatments (n = 6 in 2020 and n = 8 in 2021). Asterisks above the blue dashed indicate significant differences between the two years based on ANOVA result at P < 0.05; *** *P* < 0.001, ns; not significant.

At the harvest time, a statistically significant (p < 0.001) reduction was observed, on the average of the four water regimes in all measured yield parameters in 2020 than in 2021. Namely, in 2020, the reduction in plant height, grain yield, TGW, and the number of secondary branches per plant reached 34%, 46%, 39%, and 21%, respectively (**Figures 5A–F**). However, during both cropping seasons, the rainfed treatment showed a comparable yield to the irrigated treatment, along with the other yield components (**Figures 5A–F**). Notably, the pod number per plant and seed number per plant were not affected by the differences between the two years (**Figures 5D, F**).

The episodic drought during the V-stage and Fl-stage reduced the yield and its components in the same manner that was observed in the photosynthetic traits **(** **Figures 5A–D**). Specifically, in comparison with rainfed treatment the reduction in grain yield after a drought during V-stage and FL-stage reached 63%, 25% in 2020, and 21% and 36% in 2021, respectively **(** **Figure 5C**). Further, the reduction in TGW differed significantly between the two drought types, in 2020, and reached 32% and 10% after a drought during V-stage and Fl-stage, respectively, but in 2021 the reduction reached almost 12% for both drought types **(** **Figure 5E**). In 2020, the drought during V-stage reduced the number of secondary branches per plant by 24%; however, the statistical analysis showed that this rate was not significant because of a high standard deviation across the replicates (**Figure 5B**). Also, the pod number per plant was reduced after a drought during V-stage and Fl-stage by 59% and 30% in 2020, and 0% and 43% in 2021, respectively **(** **Figure 5D**). In the same trend, the reduction in seed number per plant after a drought during V-stage and Fl-stage reached 61.3% and 30.5% in 2020, and 0% and 36.1% in 2021, respectively **(** **Figure 5F**).

## Discussion

Owing to the expedited climate change, many scholars and breeders are pursuing identifying and screening diverse soybean cultivars to document traits backing to select drought-tolerant cultivars (Wang et al., 2018; Yan et al., 2020; Arab et al., 2022). They claimed that the plant functional traits, which are potentially linked to drought tolerance, may ensure recovery and resilience to drought under unpredictable climate change (Arya et al., 2021). Theoretically, plant functional traits, namely morphological and physiological traits, can track environmental changes and reflect the adaptive strategy of the crop to changing climate conditions (Wijewardana et al., 2018; Zhuang et al., 2020; Monteoliva et al., 2021). For empirical and robust quantification of resilience to drought, developing time series measurements is required. However, achieving this goal under field conditions entails selecting parameters that can be measured rapidly, non-destructively, and with digital techniques. Morphological traits, e.g., plant height, and some physiological traits, e.g., Chlc and ChlF ratio, and LST, meet this requirement. Further, the impact of drought on any of the plant traits depends on the timing of drought incidence. Therefore, this study attempted to explore and document for the first time the characteristics and potential resilience to drought in a drought-tolerant soybean, under field conditions, and at the V-stage and Fl-stage.

### Impact of seasonal variations of rainfall on soybean responses to drought at different growth stages

The cropping season 2020 was considered a dry season, as judged by notable low precipitation during the emergence stage (sowing-seedling), where the plants received only around 0.7 mm day^-1^ within the first 20 days after sowing (**Figure 1A**). This was below 1.3 mm day^-1^, which is considered the minimum water requirement for soybean at this stage (Norberg et al., 2010; Lamichhane et al., 2020). In addition, the total accumulated precipitation in the season, from May-September, in 2020, was also low, around 177 mm, which caused a moderate or extremely dry season, as it was classified for soybean by Pejić et al., 2011 and Schmidt et al., 2019. In contrast, in 2021, enough precipitation, almost 2.3 mm day^-1^, during the emergence stage, apparently supported the rapid growth and higher productivity in all treatments, even when drought occurred at V-stage and Fl-stage (**Figures 1A, B**, **Figures 3A–H**, and **Figures 5A-C, E**). This finding is in line with other studies that reported the importance of securing water requirements for soybean during their emergence stage to ensure well-established and successful nodulation (Norberg et al., 2010; Gajić et al., 2018; Omari et al., 2022). In this context, this result reveals that water availability during stand establishment of soybean is essential, even if cultivars were classified as drought-tolerant. As a result, seedling vigor is influential on plant responses to the subsequent drought, as we observed in both years. To sum up, if soybean does not receive enough water for optimum emergence and establishment, irrigation in later stages may be less effective to achieve high grain yields.

### Effect of drought at different growth stages on morphological traits of soybean

Plant height is an important morphological characteristic that directly informs a crop’s overall growth and development (Chavarria et al., 2017; Dong et al., 2019; Sobko et al., 2019). Results in **Figures 3A, B** show that drought inhibited the growth of soybean in terms of plant height in contrasting patterns over the two years. Namely, in 2020, the shortest plants were observed after a drought during V-stage, whereas in 2021, the shortest soybean was observed after a drought during Fl-stage. Further, upon releasing drought, plant height did not show a recovery to reach the control plant (rainfed treatment), neither when the soybean was subjected to drought at V-stage nor Fl-stage (**Figures 4A, B**). An explanation for this phenomenon is obviously strongly related to the soil moisture condition pre-, during, and after the drought event. Specifically, in 2020, the soil moisture was low during seedling emergence, then followed by an intense and prolonged moisture deficit during simulating the drought event, and finally, the plants received less rainfall during the recovery period at maturity (**Figures 2A, B**). All these conditions combined resulted in intensified and prolonged moisture deficits that were more harmful than when drought occurred during the Fl-stage in the same year. Upon that, it might be that the processes of cell division and cell expansion were strongly affected in the driest conditions and caused shorter plants, regardless of the plant growth stage. (Wei et al., 2018; Saleem et al., 2022). This finding is against our hypothesis and in contrast with some other views, where it was supposed that soybean can show compensation for drought effects when drought occurs at the V-stage (Gajić et al., 2018; Dong et al., 2019; Cui et al., 2021). We may relate these conflicting findings, from the different studies to the experimental type (i.e., field vs. greenhouse experiments), drought duration (i.e., short vs. long-term), and drought intensity (i.e., mild vs. severe). In addition, the threshold of soil moisture content for each studied cultivar may cause permanent damage to the plant.

However, the suppressed growth after a drought during the Fl-stage is consistent with our hypothesis and in line with other studies that reported the potentially irreversible negative effects on soybean in response to drought at the Fl-stage (Cui et al., 2021; Wang et al., 2022). This finding points out that soybean response to drought and recovery dynamics to achieve drought resilience is not necessarily dependent on the growth stage. Instead, pre-, and post-drought conditions, drought intensity, and duration may be more relevant to drought resilience, as was proved in multiple previous studies (Dong et al., 2019; Yan et al., 2020; Saleem et al., 2022). In comparison with previous studies that reported soybean recovery and resilience to drought (Wei et al., 2018; Dong et al., 2019; Yan et al., 2020; Cui et al., 2021), we indicate that their results may be not reflected in ‘real-world’ field conditions.

### Effect of drought at different growth stages on photosynthetic traits of soybean

Photosynthetic parameters, such as Chlc and ChlF ratio, are widely used to identify drought-tolerant genotypes (Dong et al., 2019; Iqbal et al., 2019; Guzzo et al., 2021; Arab et al., 2022). Specifically, studies reported that drought-tolerant crops/cultivars are able to increase or maintain Chlc and ChlF ratio under low water availability. We found similar responses for the first on the studied cultivar Acardia under specific conditions, which may explain the mechanism behind classifying this cultivar as drought-tolerant (Hofmann et al., 2019). Precisely, in 2020, a significant increase was observed in Chlc and ChlF ratio after a drought during V-stage and Fl-stage; however, the two parameters dropped during the maturity stage (**Figures 3C, E** **;** Monteoliva et al., 2021). Multiple potential mechanisms can underlie this capacity of a crop/cultivar to protect the photosynthetic apparatus, e.g., osmolyte accumulation, antioxidant defense system, scavenging reactive oxygen species, etc. (Lotfi et al., 2019; Guzzo et al., 2021; Wang et al., 2022). Of course, this needs further exploration and maybe several of these indices differ between drought-tolerant plants, despite that the ultimate difference is either to maintain or degrade Chlc at certain critical moisture or at certain critical stress times. The sudden drop in Chlc and ChlF ratio during the maturity stage is ascribed to the decrease in rainfall during this stage, posing a second spell of drought in the same season. Specifically, the soybean suffered from an extended drought of 24 and 27 days during V-stage and Fl-stage, respectively, and soil moisture of 7% (**Figures 1A**, **2A** **,** **3C, E**). Previous studies also reported a decrease in the Chlc and ChlF ratio is proportional to the intensity and duration of the drought (Buezo et al., 2019; Dong et al., 2019; Iqbal et al., 2019; Dubberstein et al., 2020; Monteoliva et al., 2021). As a consequence, no resilience was observed for Chlc and ChlF raion in 2020 (**Figures 4C, E**). In this context, we indicate drought-tolerant cultivars may display photosynthetic tolerance after one drought event, but fall when the water availability becomes limited during the recovery time. Given together, the pre- and after soil moisture conditions, drought duration may allow the plants to regulate certain physiological and biochemical changes that can support drought recovery after drought release. Therefore, improving the stability of photosynthetic induction under drought stress can be a promising strategy for crop breeding.

In 2021, In line with our hypothesis, soybean maintained Chlc and ChlF ratio at a balanced level with the rainfed treatment after a drought during V-stage (**Figures 3D, F**), resulting in partial recovery for ChlF ratio and Chlc that reached 35% and 40%, respectively. This finding is an indicator of adaptability to drought which may be sensitive to the stress level, duration, and post-drought moisture, in this case, at 9% soil moisture within 14 days and 12% soil moisture in the post-drought period (**Figures 4C, F** **;** Wang et al., 2018 **;** Dong et al., 2019). This finding implies that, under some specific conditions, if soybean cannot ensure conserving enough water in the leaves that is adequate to protect the functionality of the photosynthetic organs, permanent damage to the photosynthetic apparatus is expected (Wang et al., 2018; Sakoda et al., 2021; Saleem et al., 2022). Specifically, during the recovery period, photosynthetic physiological parameters were affected by the interaction of growth stage, soil moisture, and drought duration (**Figures 1**–**3**; Chen et al., 2016; Trueba et al., 2019; Dubberstein et al., 2020; Arab et al., 2022). Our findings denote that the photosynthetic recovery of the studied cultivar may require a threshold of soil moisture of 9-12% to secure partial resilience.

Surviving the studied cultivar under extremely low soil moisture conditions during Fl-stage, specifically, 1% of the rainfed treatment, is asserting being a drought-tolerant cultivar. Observing no photosynthetic recovery after drought at FL-stage, in both years, apparently signifies irreversible changes to the physiological process in the plant, because of not meeting the threshold of soil moisture at this stage (Wang et al., 2022). An interpretation of the response is that prolonged limited water availability or intensive drought conditions cause dehydration-induced injury consequences, such as damage to the chloroplast because of the harmful reactive oxygen species (Trueba et al., 2019). This process is immediately followed by a decline in the ChlF ratio and chlorophyll content in plant tissues (Wang et al., 2018; Dubberstein et al., 2020).

### Effect of drought at different growth stages on leaf thermal traits of soybean

In our study, on average for both years, drought during V-stage and Fl-stage increased LST significantly during recovery by 21% (**Figures 3G, H**). An explanation for this finding is due to water shortage under drought, a reduction may have occurred in the stomatal conductance and transpirational cooling, which induced increasing the leaf temperature (Ismanov et al., 2019; Trueba et al., 2019; Deva et al., 2020; Sakoda et al., 2021). We confirm this explanation from our own measurements that we conducted only in August 2021, during the recovery period, where drought at V-stage and Fl-stage contributed to a significant reduction in transpiration, stomatal conductance, and net photosynthesis (**Supplementary Figure S5**). Strikingly, by the end of the season, no recovery was observed in LST, meaning that the plants were incapable of cooling down even when soil moisture was comparable to the irrigated and rainfed treatments. This result may imply the potential occurrence of a permanent change in one or many physiological or biochemical functions after reaching a specific threshold of leaf temperature above the optimum, leading to impairing the reopening of the stomata (**Figures 4G, H**).

This finding may reveal a leaf thermal-specific trait of the studied cultivar, which is the high sensitivity of stomata during and post-drought (Wang et al., 2018; Deva et al., 2020; Zhuang et al., 2020). It may unveil a potential mechanism behind describing the considered cultivar as a drought-tolerant that is established in the leaves *via* closing of the stomata to secure conserving water when drought occurs. Another potential mechanism in this cultivar is the potential presence of specific protection systems that protect the photosynthetic apparatus, e.g., chloroplast thylakoid membranes, from being damaged when the LST is elevated. This potential is proved by the amounts of ChlF ratio and Chlc that increased in 2020 or were maintained during recovery time and ended with a reduction of 7-11%, in comparison with the control treatments (**Figures 3C–F**). According to that, we can attribute the inhibition of photosynthesis after drought at any growth stage of degradation or downregulation of photosynthetic enzymes, e.g., Rubisco, more than direct damage in PSII. This finding is in line with previous studies reported, that in C3 plants, e.g., soybean, elevating leaf temperature is directly affecting the thermal stability of photosynthetic enzymes, resulting in inhibition of photosynthesis (Iqbal et al., 2019; Dubberstein et al., 2020; Moore et al., 2021). Taken together, for the studied cultivar, the thermal recovery was not possible after the plant experienced drought for a duration of 14-24 and 27-29 days during V-stage and Fl-stage, respectively. Therefore, further studies need to evaluate the potential recovery of a thermal trait at shorter durations to determine the critical period when drought effects become irreversible.

### Effects of drought at different growth stages on the yield of soybean and its components

Overall, on an average of all treatments, the observed grain yield in our experiment was higher in 2021 than in 2020 by 46% (**Figure 5C**). Further, the yield in 2021 was higher than the observed average yield of different soybean cultivars that have been investigated in multiple field studies conducted at the same research station in northeastern Germany (Reckling et al., 2020; Karges et al., 2022; Omari et al., 2022; Reckling, 2022). This finding confirms that the studied cultivar was well adapted to the location of the study, but can also be related to the smaller plot size used in our controlled experiment (Rebetzke et al., 2014). Specifically, under rainfed conditions, the grain yield was 2.9 t h^-1^ in 2020 (the year with less rainfall) and 4.8 t h^-1^ in 2021 (the year with more rainfall). Interestingly, the induced drought during V-stage and Fl-stage in 2020 resulted in grain yields of 1.1 t h^-1^ and 2.2 t h^-1^, respectively. While in 2021, drought during V-stage and Fl-stage resulted in grain yields of 4.1 t h^-1^ and 3.3 t h^-1^, respectively. Obviously, the low yield in 2020 was likely due to lower precipitation than in 2021, along with the high solar radiation in 2020, 19.2 MJ m^-2^ (**Figures 1A, B**), that exceeded the optimal level for soybean in Northern Europe (17.5 MJ m^-2^; Boulch et al., 2021). Taken together, the productivity of the studied cultivar is promising for growing soybean in the future in Northern Europe, even if there is a potential for periodic drought events during the V-stage or the Fl-stage.

Previous research affirmed that irrigation is necessary to support soybean growth and grain yields (Gajić et al., 2018; Boulch et al., 2021; Pinnamaneni et al., 2021; Karges et al., 2022), however, irrigation did not differ significantly from the rainfed treatment, which may be because of the balanced precipitation pattern in the two years studied. This explanation also agrees with the fact that cumulative drought, e.g., reduced precipitation during the growing season, may trigger the stress memory in the plant, allowing it to adjust its metabolism and function to withstand drought (Elsalahy et al., 2020). Noteworthy, in the year with more rainfall (2021), a non-significant negative impact of irrigation was observed on the grain yield and TGW, representing a 10% and 8% reduction, respectively. The reason for this response is likely the lodging and poor conditions in the soil, e.g., limited aeration, after exceeding a certain level of the water requirements for soybean.

In addition, our findings confirmed previous research (Wijewardana et al., 2018; Sobko et al., 2019; Saleem et al., 2022) that, in legumes, plant height is a growth trait that can be reasonably correlated to grain yield and its components (**Figures 5A–F**). Regardless of treatment, observing shorter soybean was always proportional to lower grain yield in both growing seasons (**Figures 5A, C**). Specifically, in 2020, the overall reduction in grain yield was mainly attributed to the overall dry conditions this year than to 2021 (**Figures 2A, B** and **Figures 5A, C**). Particularly, soil moisture deficit was pronounced in 2020, during the period of pod filling until full maturity, therefore showing a significant negative impact on plant growth, the final yield, and yield components (**Figures 5A–F** **;** Wijewardana et al., 2018; Ismanov et al., 2019).

The episodic drought at the V-stage and Fl-stage significantly affected grain yields and TGW. This confirms the sensitivity of soybean yield to water deficit at different growth stages (**Figures 5C, E**; Wijewardana et al., 2018). The number of secondary branches per plant differed significantly between the two years; however, did not differ between the treatments (**Figure 5B**). This response implies that the number of secondary branches per plant might be controlled by the traits of the genotype and the water availability during the early stages of plant growth. Drought events in later stages did not alter the number of secondary branches. This finding is in contrast with Gao et al., 2020 and Arya et al., 2021, who reported a reduction in branching numbers in soybean in response to drought, which is likely attributed to differences in the studied cultivars and environmental conditions. Regardless of the growth stage, soil moisture reduced pod number per plant and seed number per plant significantly, as observed during V-stage in 2020 when soil moisture was 7% for 24 days, and Fl-stage in 2021 when soil moisture was 1% for 27 days (**Figures 2A, B** **;** **Figures 5D, E** **;** Wijewardana et al., 2018). Taken together, the overall effect of weather data in 2020 constrained the photosynthetic function and grain filling process, which resulted in a lower yield in 2020 than in 2021 (Chavarria et al., 2017; Ismanov et al., 2019; Pinnamaneni et al., 2021).

## Conclusions

Episodic drought is a prolonged period of no precipitation projected to occur more frequently in the future in Central Europe, which may affect randomly soybean at any growth stage. Hence, crop scientists and breeders asserted that drought-tolerant cultivars are adapted to different conditions, therefore, can be drought-resilient. To prove this potential under field conditions, there is a need to quantify soybean resilience to drought by using rapid, non-destructive, and repeated measurements to understand the dynamics of resilience and its reflection on the grain yield. In the present study, the morphological and physiological characteristics (plant height, Chlc, ChlF ratio, and LST) of a drought-tolerant cultivar of soybean were estimated for the first time. Plant height reduced significantly whether drought occurred at V-stage or Fl-stage, but in contrasting patterns in two exceptional years. This finding reveals that the drought effect on the considered cultivar was not dependent on drought timing, but was influenced by the pre- and post-soil moisture conditions along with the duration and intensity of drought. The Chlc and ChlF ratio slightly increased or was maintained after drought release, ultimately decreasing by 7% and 11%, respectively. Importantly, when drought was short and less intense, there was a potential for photosynthetic recovery by 35% for ChlF ratio and 40% for Chlc, implying a partial recovery of the photochemical apparatus. In contrast, LST increased significantly because of drought and was not restored to the physiological functional ranges after drought release, neither during the V-stage nor the Fl-stage.

Noteworthy, in the year with sufficient rainfall, the grain yield of the studied cultivar was 5.2 t h^-1^ and reached 4.1 t h^-1^ when Chlc recovered partially. Hence, the recovery of only the Chlc trait was not strongly reflected in the growth, and yield of soybean. Thus, the recovery in the leaf thermal trait would likely be essential for the resilience of soybean to drought, otherwise, the drought-tolerant cultivars of soybean would consider partially resilient. Further studies is needed to identify morpho-physiological traits of drought-tolerant cultivars and the underlying physiological mechanisms to understand deeply the potential extent of drought resilience in environments threatened by drought, e.g., Northern Europe. For breeding programs, when the identification of certain traits relevant to drought resilience is successful and affirmed in different genotypes, it would go a far way toward developing drought-resilient cultivars of soybean. To this end, the current study contributes to breeding programs by highlighting the importance of improving the recovery of investigated traits, specifically, the thermal temperature to build resilience to drought under the current and future climate scenarios in Northern Europe.

## Data availability statement

The original contributions presented in the study are included in the article/**Supplementary Material**. Further inquiries can be directed to the corresponding author.

## Author contributions

MR conceived and designed the experiments, conducted the experiments, and collected the data. MR provided the facilities and advised on the preparation of materials. HE and MR wrote the manuscript, read and edited the manuscript. HE performed the statistical tests. Both authors approved the final manuscript.

## Funding

This work was financed by the SusCrop-ERA-NET project LegumeGap (Grant No. 031B0807B). MR was funded by the Deutsche Forschungsgemeinschaft (DFG, German Research Foundation) – 420661662).

## Acknowledgments

For support with data collection and processing, we thank Kathleen Karges, Gunhild Rosner, Christoph Möller, Dr. Dietmar Lüttschwager, Lars Richter, Mosab Halwani, and all staff at the ZALF research station in Müncheberg.

## Conflict of interest

The authors declare that the research was conducted in the absence of any commercial or financial relationships that could be construed as a potential conflict of interest.

## Publisher’s note

All claims expressed in this article are solely those of the authors and do not necessarily represent those of their affiliated organizations, or those of the publisher, the editors and the reviewers. Any product that may be evaluated in this article, or claim that may be made by its manufacturer, is not guaranteed or endorsed by the publisher.
