# Supplementary material for: Soybean resilience to drought is supported by partial recovery of photosynthetic traits
Source: Front Plant Sci. 2022 Oct 20;13:971893. doi: 10.3389/fpls.2022.971893 (PMC9632626; doi:10.3389/fpls.2022.971893)
Supplement: Supplementary file 1 [file DataSheet_1.pdf]

Supplementary Material

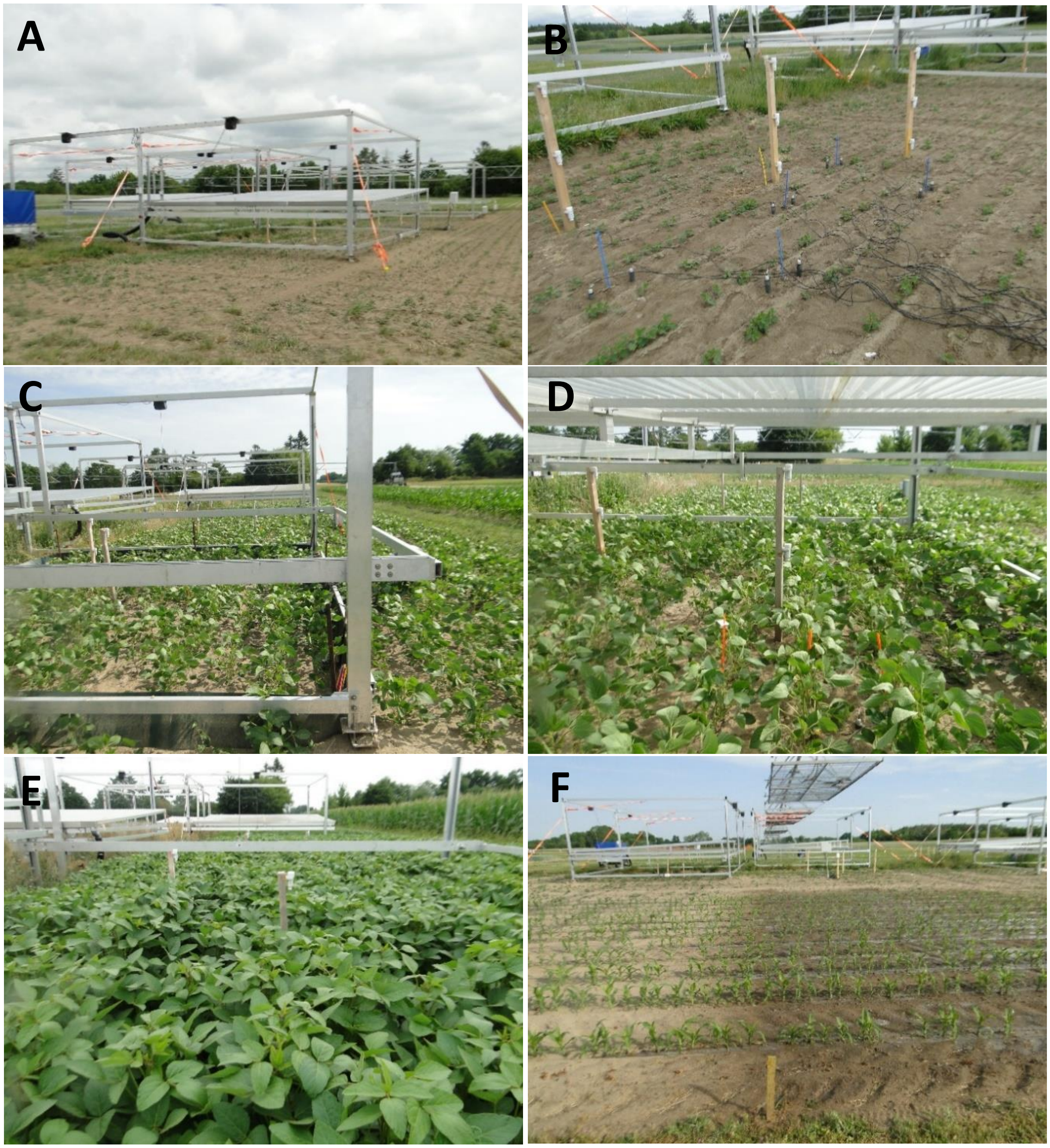

FIGURE S1 | Photographs showing the installation of the rainout-shelter (A), sensors to measure soil and air moisture and temperature on 5th June 2020 (B); the rainout-shelter in use, open in the front (after it was closed during vegetative stage) and closed in the back at flowering stage (C), one plot in focus with the roof closed at the flowering stage (D) on 29th June 2021; the rainout-shelter in use, open in the front (after it was closed during vegetative stage) and closed in the back at flowering stage on 21st July 2021 (E); and a test of the irrigation system on 9th June 2020 (F). Pictures by G. Rosner/ZALF.

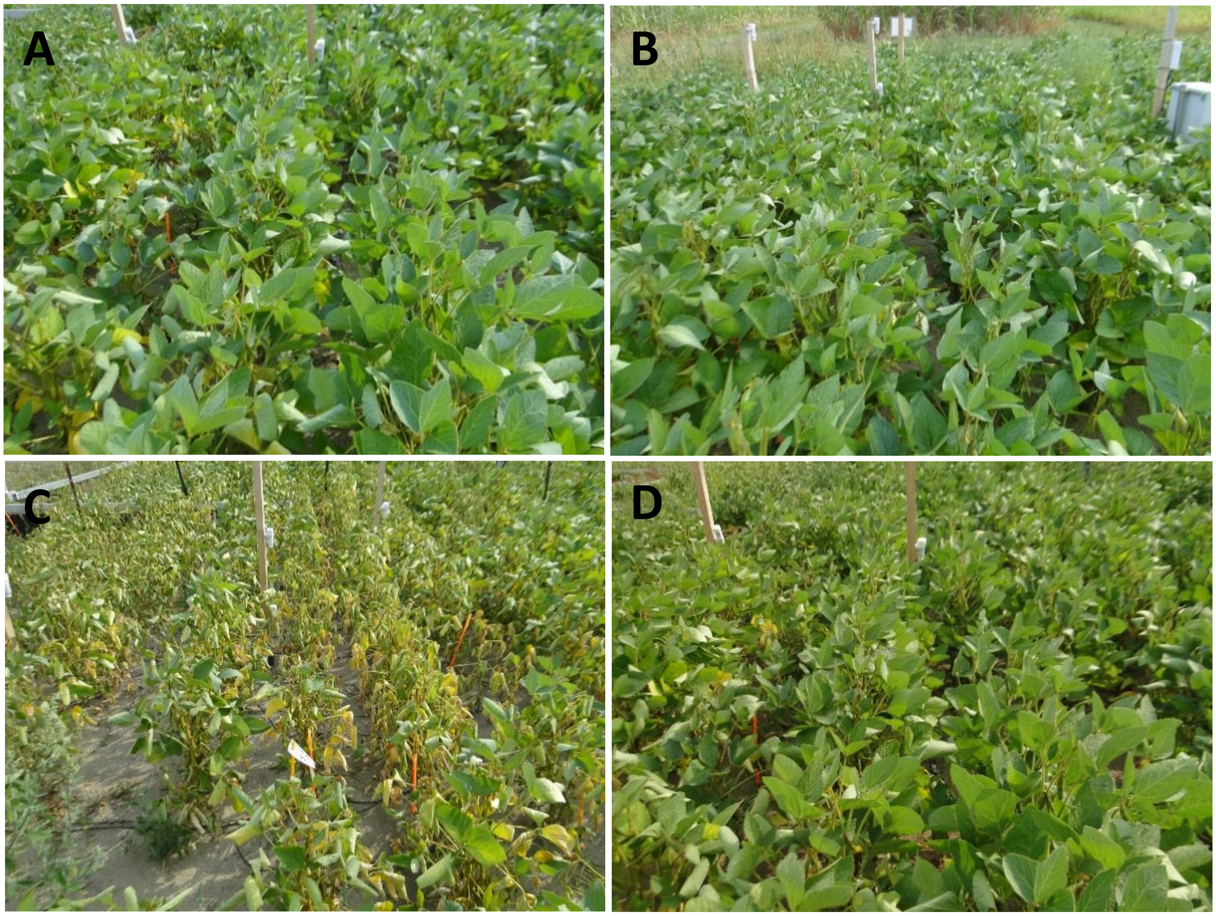

FIGURE S2 | Soybean plots after induced drought stress on 10th August 2020 and the treatments irrigated (A), rainfed (B), drought during vegetative stage (C) and drought during flowering stage (D). Pictures by G. Rosner/ZALF.

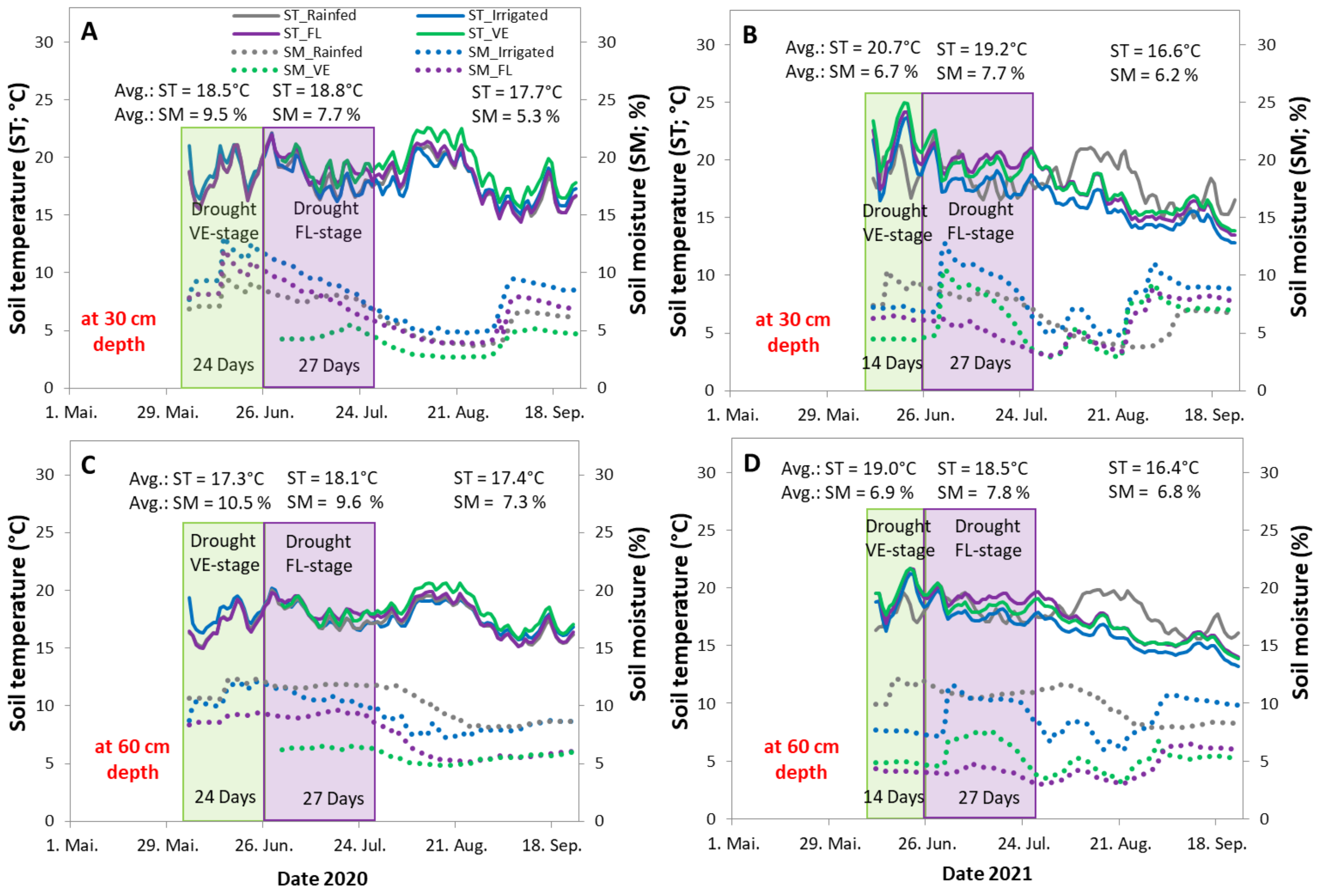

FIGURE S3 | Soil temperature (left axis) and soil moisture (right axis) at 30 and 60 cm height from the beginning of May until the end of September in 2020 (Panels A and C) and 2021 (panels B and D). The green shaded area indicates the duration of using a shelter above the plants at vegetative stage (V-stage) and the violet shaded area indicates the duration of using a shelter above the plants at flowering stage (Fl-stage).

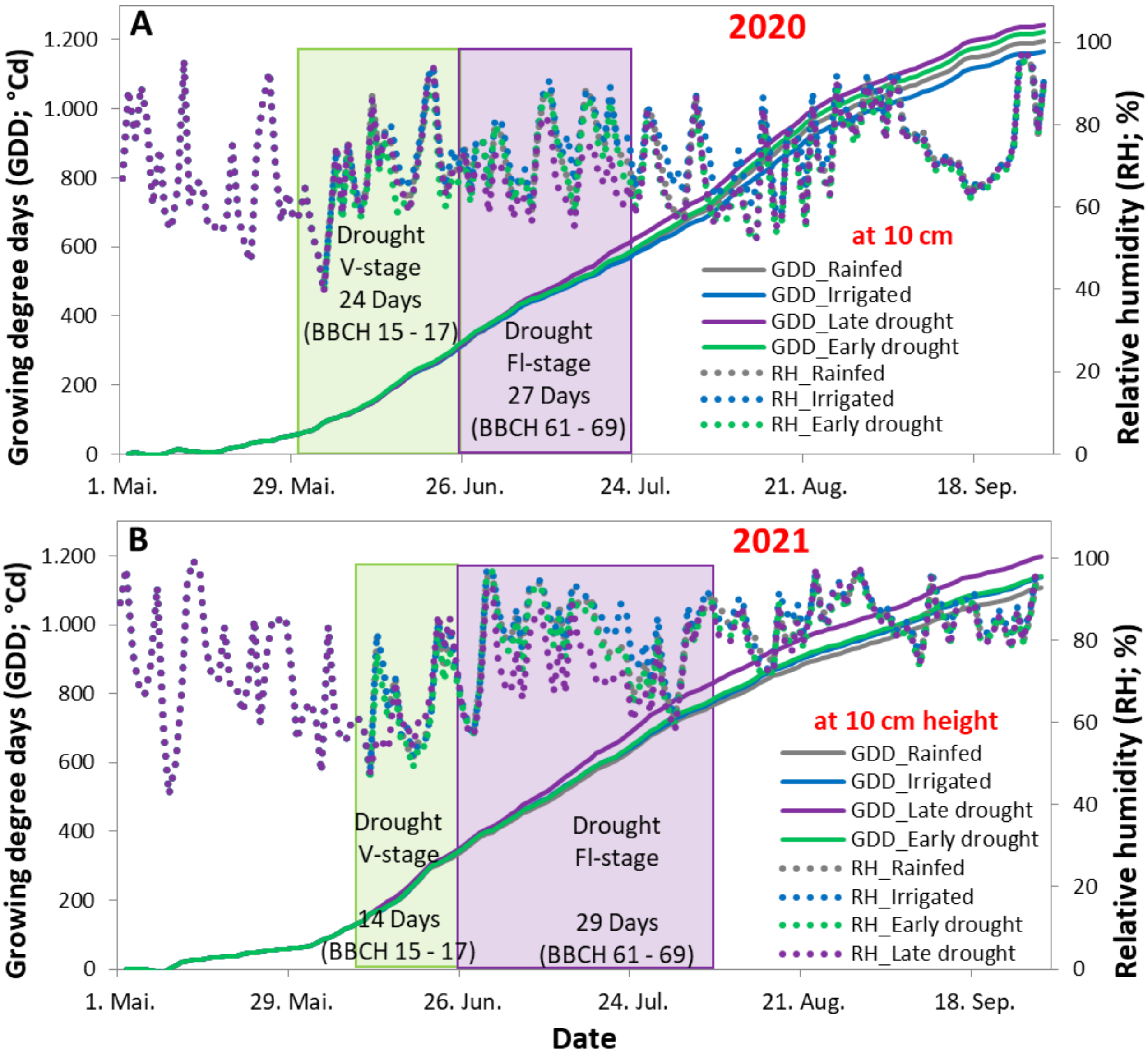

FIGURE S4 | Growing degree days (GDD; left axis; panels A and B) and relative humidity (right axis; panels A and B) from the beginning of May until the end of September in the season 2020 (A) and 2021 (B), the control treatment (rainfed; gray solid and dashed lines), the irrigated treatment (blue solid and dashed lines), the early drought treatment (green solid and dashed lines), the late-drought treatment (violet solid and dashed lines). The green shaded area indicates the duration of using a shelter above the plants at the vegetative stage (V-stage) and the violet shaded area indicates the duration of using a shelter above the plants at the flowering stage (Fl-stage).

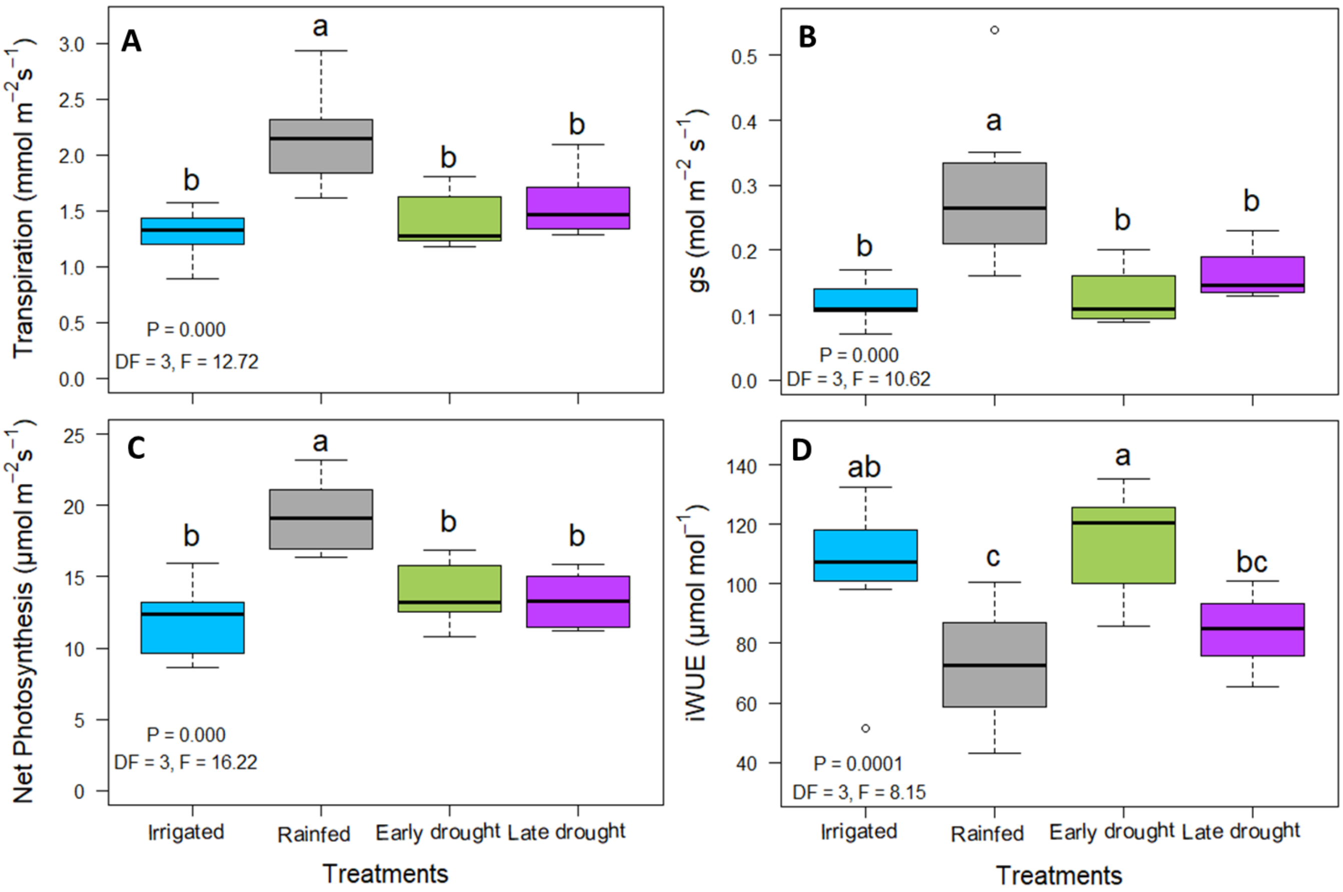

FIGURE S5 | Boxplot of photosynthetic parameters of soybean under four water treatments in 2021, transpiration (A), stomatal conductance (B), net photosynthesis (C), and intrinsic water use efficiency (D). The different letters above the boxplots represent Tukey's HSD test ($p < 0.05$) among the different treatments ($n = 8$). Photosynthetic parameters were measured on 18.08.2021 between 08:00 to 11:00 h, in a fully expanded leaf at the eighth node. The following settings of PARi = 1,000, flow = 500 µmol mol^{-1}, stomatal ratio = 0.5, and reference CO_2 concentration = 400 µmol mol^{-1} were used.

TABLE S1 | Dates and amount of irrigated water for soybean (cv. Acardia) during the two growing seasons 2020 and 2021.

Year	Date	Amount of irrigation (mm)
2020	18.06.2020	20
2020	24.06.2020	20
2020	03.07.2020	20
2020	13.07.2020	20
2020	22.07.2020	20
2020	29.07.2020	20
2020	31.07.2020	20
2020	04.08.2020	20
2020	05.08.2020	20
2020	11.08.2020	20
2020	12.08.2020	20
2020	14.08.2020	20
2020	21.08.2020	20
2020	26.08.2020	20
Sum in 2020		280
2021	17.06.2021	20
2021	23.06.2021	20
2021	15.07.2021	20
2021	22.07.2021	20
2021	05.08.2021	20
2021	17.08.2021	20
2021	18.08.2021	20
Sum in 2021		140

TABLE S2 | Model selection, based on AICc comparison, of linear mixed model (lme) describing plant height, chlorophyll florescence ratio (ChlF ratio), chlorophyll content (Chlc), and leaf surface temperature (LST) of soybean under four water treatments (Trt) at different times of crop growth (Tim) in two years (Yr).

Model	(Int)	T	Trt	Yr	T:Trt	T:Yr	Trt:Yr	T:Trt: Yr	family	correlation	weights	random	df	logLik	AICc	delta	weight	p-value
lme1	37.0	6.3	+	+	+	+	+	+	Gaussian (identity)	corAR1()	varIdent(~1| Year*Trt)	Yr/T	27	-489.4	1042.8	0	1	
lme2	37.2	6.2	+	+	+	+	+	+	Gaussian (identity)			Yr/T	19	-519.3	1081.4	38.5	0	<.0001
lme3	36.9	6.3	+	+	+	+	+	+	Gaussian (identity)	corAR1()		Yr/T	20	-519.1	1083.6	40.7	0	<.0001
lme4	37.2	6.2	+	+	+	+	+	+	Gaussian (identity)		varIdent(~1| Year*Trt)	Y	25	-624.8	1308.1	265.3	0	<.0001

TABLE S3 | The mixed ANOVA model for the effects of Year (Yr), measurement time (Tim), Treatment (Trt), and their interactions on plant height, Chlorophyll florescence ratio (ChlF ratio), Chlorophyll content (Chlc), and leaf surface temperature (LST), with time nested in year as the random factor.

Year	Explanatory variables	Response variables												
		Plant height (cm)				ChlF ratio			ChlC			LST		
		DF	F-value	P-value		F-value	P-value		F-value	P-value		F-value	P-value	
Both	Year (Yr)	1,164	204.2	<.0001	***	331.5	<.0001	***	325.0	<.0001	***	16.1	<.0001	***
	Measurement time (Tim)	1,164	408.2	<.0001	***	131.1	<.0001	***	129.3	<.0001	***	0.3	0.59	ns
	Treatment (Trt)	3,164	24.2	<.0001	***	1.1	0.386	ns	1.2	0.299	ns	0.7	0.556	ns
	Yr:Tim	1,164	21.5	<.0001	***	89.8	<.0001	***	89.9	<.0001	***	24.3	<.0001	***
	Yr:Trt	3,164	3.1	0.030	*	0.5	0.708	ns	0.6	0.639	ns	0.4	0.721	ns
	Tim:Trt	3,164	4.3	0.006	**	6.3	0.000	***	5.5	0.001	**	1.2	0.305	ns
	Yr:Tim:Trt	3,164	1.6	0.202	ns	2.0	0.112	ns	1.9	0.130	ns	0.3	0.793	ns
	Random effect: (Yr|Tim)	StdDev = 10.94				StdDev = 0.16			StdDev = 102.5			StdDev = 9.73		
2020														
	Measurement time (Tim)	1,88	184.3	<.0001	***	311.5	<.0001	***	312.9	<.0001	***	16.8	<.0001	***
	Treatment (Trt)	3,88	22.4	<.0001	***	0.2	0.898	ns	0.2	0.894	ns	1.3	0.276	ns
	Tim:Trt	3,88	5.7	<.0001	**	4.5	0.005	**	4.6	0.005	**	2.2	0.0954	ns
	Randam effect (Tim)	StdDev = 7.51				StdDev = 0.1			StdDev = 63.09			StdDev = 0.002		
2021														
	Measurement time (Tim)	1,76	218.6	<.0001	***	2.2	0.141	ns	2.0	0.157	ns	9.9	0.002	**
	Treatment (Trt)	3,76	9.4	<.0001	***	1.1	0.374	ns	1.2	0.307	ns	0.2	0.869	ns
	Tim:Trt	3,76	1.6	0.192	ns	3.8	0.013	*	3.1	0.031	*	0.2	0.920	ns
	Randam effect (Tim)	StdDev = 13.18				StdDev = 0.13			StdDev = 86.78			StdDev = 0.02		

Asterisks indicate level of significance (ns <0.1, * <0.05, ** <0.01, *** <0.001).

TABLE S4 | The mixed ANOVA model for the effects of Year (Yr), measurement time (Tim), Treatment (Trt), and their interactions on the resilience of soybean in terms of the measured plant height, Chlorophyll florescence ratio (ChlF ratio), Chlorophyll content (Chlc), and leaf surface temperature (LST), with time nested in year as the random factor.

Year	Explanatory variables	Response variables												
		Resilience for plant height				Resilience for ChlF ratio			Resilience for Chlc			Resilience for LST		
Both	**Both years:**	DF	F-value	P-value		F-value	P-value		F-value	P-value	0	F-value	P-value	
	Year (Yr)	1,52	8.3	<.0001	***	6.5	0.0136	ns	8.3	0.0058	ns	1.5	<.0001	***
	Measurement time (Tim)	1,52	0.1	<.0001	***	4.7	<.0001	***	5.0	<.0001	***	9.5	0.00326	***
	Treatment (Trt)	1,52	57.1	<.0001	***	0.5	0.464	ns	0.0	0.894	ns	20.3	3.8E-05	***
	Yr:Tim	1,52	8.0	0.0068	ns	0.6	0.433	ns	0.2	0.659	ns	2.4	0.127	ns
	Yr:Trt	1,52	1.1	0.306	ns	28.8	0.000	***	31.5	0.000	***	3.6	0.064	**
	Tim:Trt	1,52	1.3	0.259	ns	14.6	<.0001	***	11.3	<.0001	***	1.2	<.0001	***
	Yr:Tim:Trt	1,52	0.2	0.677	ns	1.1	<.0001	***	0.4	<.0001	***	0.2	0.667	ns
	Random effect: (Yr|Tim)	StdDev = 0.12				StdDev = 0.16			StdDev = 102.5			StdDev = 9.73		
2020														
	Measurement time (Tim)	1,29	4.4	<.0001	***	4.7	<.0001	***	3.5	<.0001	***	7.4	0.01082	***
	Treatment (Trt)	1,29	34.5	<.0001	***	17.3	0.000	***	12.1	0.002	***	14.5	0.00068	***
	Tim:Trt	1,29	1.1	<.0001	***	6.1	<.0001	***	5.1	0.032	*	0.3	<.0001	***
	Randam effect (Tim)	StdDev = 0.16				StdDev = 3.63			StdDev = 1.67			StdDev = 0.00		
2021														
	Measurement time (Tim)	1,23	4.4	<.0001	***	0.9	<.0001	***	1.7	0.208	ns	2.7	0.114	ns
	Treatment (Trt)	1,23	21.9	<.0001	***	12.1	0.002	**	20.0	0.000	ns	5.8	0.024	ns
	Tim:Trt	1,23	0.2	0.6879	ns	9.2	<.0001	**	6.8	0.016	***	2.7	0.113	ns
	Randam effect (Tim)	StdDev = 0.05				StdDev = 0.00			StdDev = 0.00			StdDev = 0.09		

Asterisks indicate level of significance (ns <0.1, * <0.05, ** <0.01, *** <0.001).
